# Reactivation-induced motor skill modulation does not operate at a rapid micro-timescale level

**DOI:** 10.1038/s41598-023-29963-5

**Published:** 2023-02-20

**Authors:** Jasmine Herszage, Marlene Bönstrup, Leonardo G. Cohen, Nitzan Censor

**Affiliations:** 1 School of Psychological Sciences and Sagol School of Neuroscience, Tel Aviv University, Sharet Building, 69978 Tel Aviv, Israel; 2 Department of Neurology, University of Leipzig, Leipzig, Germany; 3 Human Cortical Physiology and Neurorehabilitation Section, NINDS, NIH, Bethesda, MD USA

**Keywords:** Consolidation, Human behaviour

## Abstract

Abundant evidence shows that consolidated memories are susceptible to modifications following their reactivation. Processes of memory consolidation and reactivation-induced skill modulation have been commonly documented after hours or days. Motivated by studies showing rapid consolidation in early stages of motor skill acquisition, here we asked whether motor skill memories are susceptible to modifications following brief reactivations, even at initial stages of learning. In a set of experiments, we collected crowdsourced online motor sequence data to test whether post-encoding interference and performance enhancement occur following brief reactivations in early stages of learning. Results indicate that memories forming during early learning are not susceptible to interference nor to enhancement within a rapid reactivation-induced time window, relative to control conditions. This set of evidence suggests that reactivation-induced motor skill memory modulation might be dependent on consolidation at the macro-timescale level, requiring hours or days to occur.

## Introduction

Consolidation is a crucial process in the formation of memories, occurring after the initial encoding of information or a skill, and resulting in memory stabilization. While previously thought to be an irreversible process^1,2^, evidence from studies in rodents^3–5^, further supported by human studies^6–8^ indicates that even fully consolidated memories can become unstable again upon their reactivation. Such reactivation can result in deterioration^7–10^ or in enhancement of the memory (^11^, for a review see^12^). Similar to previous reports across multiple memory domains^13,14^, spanning from fear memory^3,4,15^ to perceptual memories^11^, evidence for such modification of memories following their reactivation was recently demonstrated in motor skill memories as well: reactivations were shown to protect memories from future interference^16^ and even induce learning of a motor skill^17,18^.

At the neural level, the post-reactivation time window is known to involve protein synthesis-dependent processes^3^, requiring hours or days to occur^19–21^. While similar timescales were reported for post-encoding consolidation as well^22–25^, it has been shown that a rapid form of consolidation occurs even in early stages of learning, at a micro-timescale of minutes^26^. Thus, while consolidated memories often show offline learning gains between sessions, such gains were evident between trials in early stages of a single encoding session, at a micro-timescale level.

**Figure 1:**
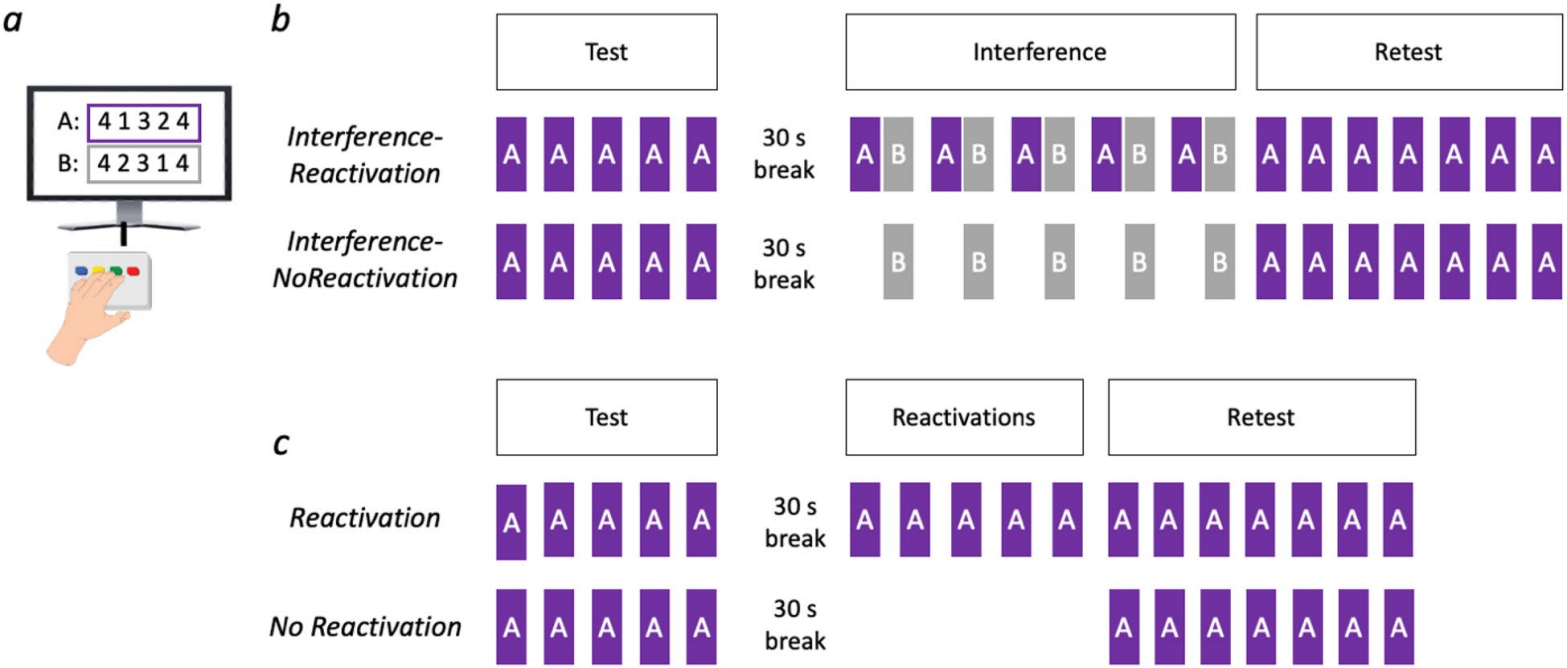
Experimental design. (**a**) The sequence tapping task required participants to tap a sequence of numbers (either sequence A: 4 1 3 2 4 or sequence B: 4 2 3 1 4) which was constantly presented on the screen during trials. (**b**) Experimental design of Experiment 1. Each purple square represents a trial of the main sequence A, and each grey square represents a trial of the new sequence B. (**c**) Experimental design of Experiment 2.

Motivated by this set of evidence, we asked whether encoded skill memories would be susceptible to modifications within a rapid 'micro-timescale' window following their reactivation. In a set of experiments, we collected crowdsourced motor sequence learning^27^ data from 459 participants recruited from the Amazon Mechanical Turk platform (MTurk), to test whether post-reactivation interference and enhancement are evident at the micro-timescale of minutes, according to the established human reconsolidation criteria^14^. Since memories were previously shown to be susceptible to interference following reactivation in the macro-timescale of 24 h between sessions^8,28^, *Experiment 1* tested whether encoded memories are susceptible to interference within a micro-timescale window induced by brief reactivations. Participants underwent rapid micro-timescale consolidation^26^ of the motor sequence task. Then, an interfering sequence was presented interleaved with brief reactivations of the original sequence. Retest was compared to a control group without reactivations (see Fig. 1b). Since reactivations were recently shown to induce learning in motor skills^17^, *Experiment 2* tested if memories can benefit from brief reactivations to produce enhanced learning gains even following rapid consolidation, and not a full, macro-timescale consolidation. Following micro-timescale consolidation, the memory was reactivated repeatedly to induce learning. Retest was then compared to a control group without reactivations (see Fig. 1c).

## Materials and methods

### Participants

Participants were recruited from the Amazon Mechanical Turk Platform (MTurk). Qualifications for registered MTurk workers to participate in the experiments were: > 95% approval rate on all previous MTurk assignments, location in the United States, right-handedness and no previous participation in any sequence learning task offered by our lab. All procedures were in accordance with a protocol approved by the Tel Aviv University Ethics committee, and all methods were performed in accordance with the relevant guidelines and regulations approved by committee. Informed consent was obtained from all participants, declaring via button press that they agree to participate and acknowledge the outline and purpose of the study, their voluntary participation, the time commitment and payment, their mandatory age above 18 years old, information on data safety and being given contact information.

Sample sizes for each experiment were estimated based on power analysis of pilot studies of motor sequence learning. Sample sizes were 230 participants for Experiment 1 (95 female, mean ± sd age 33.9 ± 7.2), and 229 for Experiment 2 (110 female, age 34.7 ± 8.8). These sample sizes represent the total number of participants after exclusion of assignments demonstrating incomplete adherence to task instructions. Participants were paid 1.5$ for the session, equivalent to > 8$ per hour. The time of the task being posted was midday on weekdays for each experiment. Tasks were posted at batches of 30–50 workers each time for administrative (monetary) reasons.

### Task

Participants practiced the motor skill learning task^29^ in which they were asked to tap, as fast and as accurate as they could, a five-digit sequence (Experiment 1: main sequence 4–1–3–2–4, new sequence 4–2–3–1–4; Experiment 2: 4–1–3–2–4; see Fig. 1a) on the numeric keys of participants’ computer keyboards with the pinky finger corresponding to button # 1, the ring finger to # 2, middle finger to # 3, and index finger to # 4. During each trial, the sequence was presented constantly on a computer screen. The task was performed with the left nondominant hand in all trials. Each trial lasted 10 s, during which feedback was provided in the form of a star displayed at the top portion of the screen, appearing immediately after each keypress regardless of correctness^17,26^. Stimuli were programmed, presented and responses recorded using the Pavlovia.org platform.

## Experimental procedure

All subjects performed identical encoding consisting of five trials lasting 10 s each with 10 s breaks in between, based on pilot experiments showing that participants reached 95% performance by trial 5. Experiment 1 tested whether early learning memories are susceptible to interference within a reactivation-induced time window. Accordingly, encoding was followed by a 30 s break, during which a countdown was consistently presented on the screen to maintain subjects’ engagement with the task. Following the break, subjects in the *Interference-Reactivations* group (N = 115) performed five reactivations trials, in which they performed the main sequence (4–1–3–2–4), each immediately followed by a trial of the new sequence (4–2–3–1–4, 5 new sequence trials in total), while subjects in the *Interference*-*NoReactivations* group (N = 115) performed the five new sequence trials with 10 s breaks in between, without reactivations. Immediately following these trials, all participants completed a retest consisting of seven trials of the main sequence (see Fig. 1b). Experiment 2 tested if early learning memories can benefit from brief reactivations to produce enhanced learning gains. Accordingly, participants in the *Reactivations* group (N = 127) performed 5 reactivations trials following the 30 s break and encoding of the skill memory, followed by a retest identical to experiment 1, while participants in the *NoReactivations* group (N = 102) performed encoding and retest without reactivations, and with a break of the same length as the duration between test and retest for the *Reactivations* group (120 s; see Fig. 1b). Of note, the number of trials used in test and retest sections was identical in both Experiment 1 and Experiment 2 to maintain consistency throughout the study.

### Data analysis

Behavioral data were analyzed with Matlab R2019b and SPSS statistics 27. Due to the experimental setting offered via crowdsourcing platforms, data registered from each participant were checked for correct implementation and adherence to task instructions. Incorrect implementation of instructions or adherence was defined by (i) completion of the task with the right hand (documented by given answer to question after the task), (ii) completion of only one sequence repetition except for trial 1 (in both experiments) and reactivation trials in Experiment 1, (iii) keypresses consistently different from instructed sequence, (iv) response times until the first key tap longer than 2 s, indicative of lack of attention to the screen, (v) no registered responses during at least one trial. Subjects with incorrect implementation (according to at least one of the above) were excluded from analyses.

To test for baseline differences between groups, a repeated measures ANOVA was conducted with the five test trials as a within subject factor, and group as a between subject factor. Test–retest improvements were evaluated with a repeated measures ANOVA with two performance levels (mean 5 test trials, mean 7 retest trials), and group as a between subject factor, Bonferroni corrected. In experiment 1, to test for gradual improvements in the new sequence, as well as gradual decrease in performance of the original sequence during reactivations, two repeated measures ANOVA were conducted with five trials (5 new sequence trials, or 5 reactivation trials) as a within subject factor, Bonferroni corrected. Each null result in an ANOVA test was further confirmed with a Bayesian approach, by calculating the Bayes factor of comparing total learning across the two conditions. Bayesian analyses were performed with JASP version 0.16.

## Results

**Figure 2:**
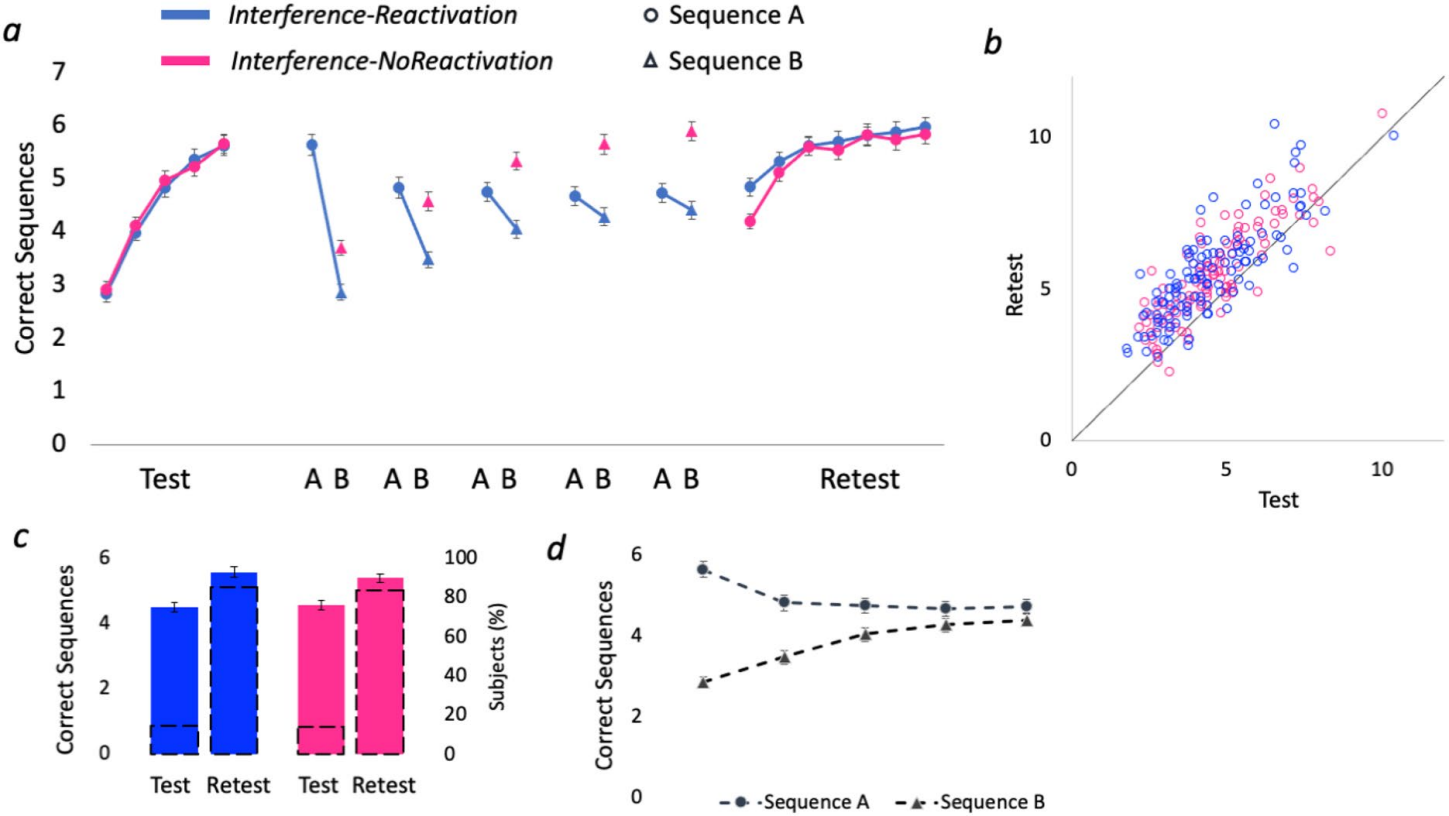
Micro-timescale reactivations do not enhance memory interference. (**a**) Single-trial performance (*Interference-Reactivations* group in blue and *Interference-NoReactivations* group in pink). (**b**) Test versus retest single-subject performance presented in a scatterplot along a unit slope line (y = x) where each point reflects a participant^11,16,30^. Data accumulating above the unit line reflect subjects who improved from test to retest, expressing less interference, while data points below the line indicate degraded retest performance, expressing stronger interference. (**c**) Colored bars reflect the mean performance in test and retest sessions (corresponding to the left y-axis), dashed black bars (corresponding to the right y-axis) reflect the percentage of participants on each side of the unit slope line in (**b**). (**d**) Mean performance of the *Interference-Reactivations* group in both sequences executed alternatingly (main sequence A as circles, interfering sequence B as triangles). Error bars represent SEM.

To test whether memories express increased susceptibility to interference during early learning within micro-timescale reactivation-induced time windows, Experiment 1 compared the effect of interference in motor sequence learning between two groups who experienced interference either with or without reactivations. Both *Interference-Reactivation* and *Interference-NoReactivation* groups first performed five trials of skill acquisition. A repeated measures ANOVA with five test trials and two group levels showed no baseline differences between the groups (no group effect F_1,228_ = 0.07, p = 0.79 and no group × trial interaction F_4,912_ = 0.80, p = 0.52; see Fig. 2a), with a complimentary Bayesian analysis confirming that there was substantial support in favor of the null hypothesis of no difference between groups relative to the alternative hypothesis for the group effect (BF_01_ = 7.10) and the group x trial interaction (BF_01_ = 127.31). Test–retest improvements showed a main effect for time (F_1,228_ = 207.69, p < 0.001), but no effect for group (F_1,228_ = 0.11, p = 0.74; BF_01_ = 3.17) nor a significant interaction (F_1,228_ = 3.40, p = 0.07; see Fig. 2b,c), suggesting that micro-timescale reactivation-induced time windows did not modulate the susceptibility to interference at early stage of skill learning. Of note, while retest performance was not significantly different between groups (F_1,228_ = 0.76, p = 0.39), post-hoc pairwise comparisons showed that the first retest trial was better in the *Interference-Reactivation* group, compared to the *Interference-NoReactivation* group (t_228_ = 2.96, p = 0.003). However, this difference diminished in the following retest trials and was not significant in any of the following retest trials (significance level: p = 0.37, p = 0.97, p = 0.53, p = 0.95, p = 0.64, p = 0.58 for trials 2–7 respectively; Bonferroni corrected).

Of note, while performance in trials of the new memory showed gradual improvements (F_4,456_ = 40.42, p < 0.001, Greenhouse–Geisser corrected; see triangles in Fig. 2d), performance in the reactivation trials showed gradual decrease (F_4,456_ = 12.54, p < 0.001, Greenhouse–Geisser corrected; see circles in Fig. 2d), suggesting that in early stages of learning, two memories can be encoded in parallel, converging performance to a shared similar level.

**Figure 3:**
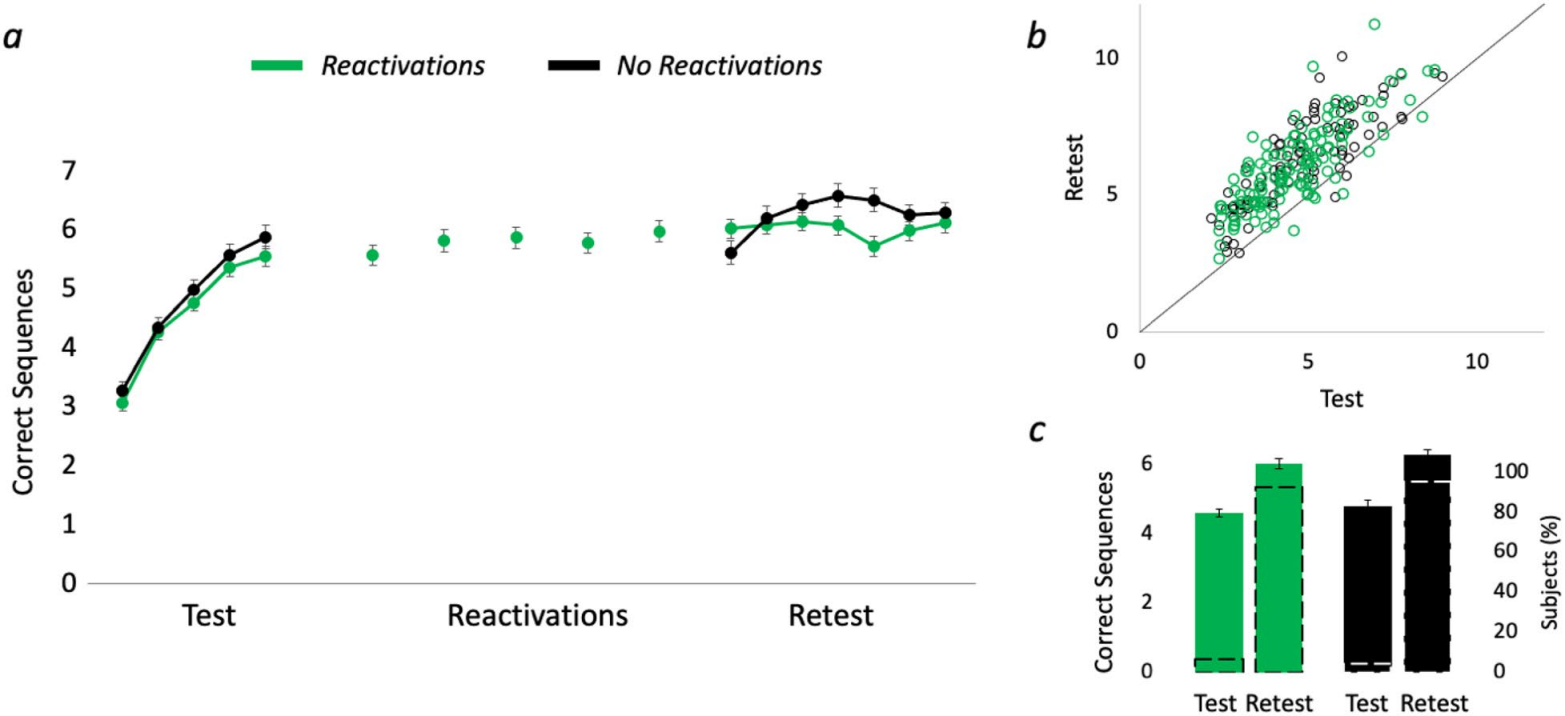
Micro-timescale reactivations do not induce learning. (**a**) Single-trial performance (*Reactivations* group in yellow and *NoReactivation* group in black). (**b**) Test versus retest single-subject performance presented in a scatterplot along a unit slope line (y = x) where each point reflects a participant^11,16,30^. Data accumulating above the unit line reflect subjects who improved from test to retest, expressing learning gains, while data points below the line indicate degraded retest performance. (**c**) Colored bars reflect the mean performance in test and retest sessions (corresponding to the left y-axis), dashed black bars (corresponding to the right y-axis) reflect the percentage of participants on each side of the unit slope line in (**b**). Error bars represent SEM.

To further investigate whether reactivation-induced skill modulation operates at a micro-timescale level, Experiment 2 tested whether such immediate reactivations of the memory induce learning, as recently reported between-days^17^. Thus, Experiment 2 maintained similar test and retest as in Experiment 1, with either 5 reactivations trials (*Reactivation* group) between test and retest, or without reactivations (*NoReactivation* group). A repeated measures ANOVA with 5 test trials and 2 group levels showed comparable baseline performance across groups during acquisition and test trials (no group effect F_1,227_ = 1.16, p = 0.28, BF_01_ = 4.16 and no group x trial interaction F_4,908_ = 0.44, p = 0.78, BF_01_ = 155.20; see Fig. 3a). Test–retest improvements showed a main effect for time (F_1,227_ = 538.91, p < 0.001), but no effect for group (F_1,227_ = 1.42, p = 0.24, BF_01_ = 2.51) nor a significant interaction (F_1,227_ = 0.10, p = 0.76, BF_01_ = 4.71; see Fig. 3b,c), suggesting that micro-timescale reactivation-induced time windows did not induce learning gains.

## Discussion

In a set of crowdsourced experiments, with data collected from hundreds of participants, this study examined whether reactivation-induced modulation of motor skill memory operates at a micro-timescale level. To address this question, two main behavioral aspects of memory modulation were tested: memory interference and performance enhancement. Based on multiple studies reporting modified effects of memory interference if a new memory is presented during the reactivation-induced time windows^8,16,28^, Experiment 1 tested whether interference with reactivated skill memory is enhanced immediately following encoding. However, results showed that the effect of interference was comparable regardless of whether the new memory was reactivated or not. In addition, since memory reactivation was recently reported to induce learning in motor skills^17,18^, Experiment 2 tested whether uninterrupted reactivations following early skill learning enhance performance. Results showed that these reactivations did not enhance skill performance, with performance at retest comparable to the *NoReactivations* group who did not undergo reactivations between test and retest. In sum, these results show that memory modulation effects reported in both human and animal studies, do not apply at the micro-timescale level, and thus do not increase the susceptibility of memories to modulations in early stages of learning.

Interestingly, rapid consolidation was reported to occur in early stages of motor skill learning, during rest periods between trials, lasting only 10 s^26^. Moreover, a recent study suggested that micro-timescale consolidation might be induced by fast neural replay occurring during wakeful rest periods between trials^31^. Even though consolidation is evident at the micro-timescale, the current study did not find evidence for an analogous form of rapid memory modulation occurring at the micro-timescale level. The reason for this might stem from the state of the memory trace itself. Consolidation processes transform unstable newly acquired memories to a stable state, in which they will be less prone to modifications, while following memory reactivation, memories are transformed from a stable state back to an unstable state. Thus, since consolidation affects unstable memories, it may be effective for newly acquired skills at early stages of learning, but reactivation-induced modulation, operating on stable memory traces, might not be effective in early stages of learning, and therefore does not operate successfully in a rapid form at the micro-timescale level.

When a second skill memory was presented during the rapid reactivation-induced time windows in Experiment 1, performance of both memories gradually converged to a common level, which was better than the starting level of the new memory, but worse than the starting level of the original memory. This notion might suggest that in early stages of learning, when the memory is still flexible enough, two memories can be acquired simultaneously, with a small detriment in performance of the original memory. Moreover, the convergence of performance towards a similar level of skill execution might imply that these memories are stored in a shared manner in the brain as a single merged memory trace, consistent with the concept of neural engrams^32^, which were shown to overlap when learning two linked memories^33^.

These findings may provide additional evidence for the long-known discussion on the fundamental dissociation between consolidation and reconsolidation, occurring during reactivation-induced time windows^34^, to be further tested in future research. A number of studies have tested the molecular differences between the two, with some studies reporting similarities at the molecular level of the underlying mechanisms^3,35^, and some studies reporting differences (for example^36–39^). In line with these studies, the results of the current study point to a dissociation between consolidation and reconsolidation, with a behavioral separation between consolidation, that indeed occurs at a rapid form within minutes^26,40^, and reconsolidation that does not operate in such a micro-timescale. Nevertheless, it should be noted that the current study is limited to specific reactivation and interference protocols, based on previous studies. Future studies could further investigate reactivation-induced skill modulation via alternative protocols. For example, while interference in Experiment 1 was performed by interleaved trials of both skill memories^16^, future studies could induce interference in blocked practice^41,42^ following single-trial reactivation and by incorporating a prediction-error^43^ as done in additional memory domains. In addition, studies can evaluate reactivation-induced skill modulation by testing interference between hands instead of alternating sequences^12,44^.

In sum, the findings of the current study provide robust evidence that contrary to the regular timescales of reactivation-induced memory modulation, interference (Experiment 1) and learning (Experiment 2) are not enhanced within reactivation-induced time windows. Thus, findings of both experiments converge to suggest that reactivation-induced modulation does not operate at a micro-timescale level. Revealing the boundaries and time frames of memory modulation can have implications on future strategies geared to modulate learning and memory.

## Data Availability

The datasets generated and analyzed during the current study are publicly available at https://osf.io/mrh9d/. This manuscript was deposited as a preprint in PsyArXiv under license CC-By Attribution 4.0 International: https://doi.org/10.31234/osf.io/8da5f.
